# Genomic sequencing of recent rabbit hemorrhagic disease virus 2 strains from diseased European rabbits supports a novel incursion into Washington State, USA

**DOI:** 10.1128/mra.00516-26

**Published:** 2026-10-22

**Authors:** Brandi K. Torrevillas, Maria Jenckel, Azeza M. Falghoush, N E. Neely, Ryan Oliveira, Amber Itle, Ben Smith, Kevin R. Snekvik, Thomas B. Waltzek

**Affiliations:** 1 Washington Animal Disease Diagnostic Laboratory, Washington State University 6760, Pullman, Washington, USA; 2 Department of Veterinary Microbiology and Pathology, College of Veterinary Medicine, Washington State University 6760, Pullman, Washington, USA; 3 Biosecurity, Commonwealth Scientific and Industrial Research Organization, Acton, Australian Capital Territory, Australia; 4 Animal Health Program, Washington State Department of Agriculture, Olympia, Washington, USA; Katholieke Universiteit Leuven, Leuven, Belgium

**Keywords:** rabbit hemorrhagic disease virus, *Lagovirus europaeus*, European rabbit, *Oryctolagus cuniculus*, *Caliciviridae*

## Abstract

Five feral European rabbits found dead in Washington state tested positive for rabbit hemorrhagic disease virus 2 (RHDV2) by qPCR. Genomic sequencing revealed a nearly identical RHDV2 strain present in the rabbits. The RHDV2 strain was determined to be divergent from previous United States detections, suggesting a novel RHDV2 incursion.

## ANNOUNCEMENT

Leporid caliciviruses belong to a single species (*Lagovirus europaeus*) segregated into two genogroups: genogroup I (GI) predominantly infecting rabbits and genogroup II (GII) infecting hares (1, 2). Lagoviruses possess ssRNA genomes organized into two ORFs: ORF1 encodes a large polyprotein that is cleaved into non-structural (NS) proteins and the major capsid protein, and ORF2 encodes the minor structural (S) protein. Lagoviruses are expressed using the formula: [RdRp genotype]P-[capsid genotype] (3–5). Here, we report the detection of a RHDV2 (GI.3P-GI.2) strain in Washington (WA) in 2024, genetically distinct from a RHDV2 (GI.4aP-GI.2) strain detected in WA the prior year (6).

In 2024, frozen European rabbit (*Oryctolagus cuniculus*) cadavers from mortality events in San Juan and King counties, WA, were submitted to the Washington Animal Disease Diagnostic Laboratory. Two of five examined specimens underwent full necropsy and histopathology. Both rabbits had hepatocellular necrosis, and one had fibrinous splenitis, both histologic lesions seen with RHDV2 infection (7). Livers from each rabbit were homogenized in Dulbecco’s Modified Eagle Medium at 1% (w/v) by glass bead disruption. Fifty microliters of each homogenate was clarified (9,000× *g* for 2 min), then extracted using a MagMAX-96 Viral RNA Isolation Kit (Applied Biosystems). All RNA extracts tested positive for RHDV2 by qPCR (8). Double-stranded cDNA was prepared for each RNA extract using tagged random octamers as previously described (9), and these cDNAs were used as templates for generating sequencing libraries with an Illumina DNA Prep Library Kit (Table 1). After trimming and filtering, paired-end reads were mapped to RHDV strain FRG (GenBank accession NC_001543) in CLC Genomics Workbench v25.0.1 using default settings. Consensus sequences were extracted from the mapping using a minimum coverage threshold of 10× and ambiguity codes for conflict resolution with 0.1 noise/10× depth threshold. The WA/2024 genomes were annotated by homology from the whole-genome alignment using the annotated FRG genome for reference. The resulting RHDV2 draft genomes included complete ORF1 and ORF2 coding sequences. The NS and S regions of the five new RHDV2 genomes (SJ1-4, KC1) were aligned with representative RHDV2 sequences, including all available North American (NA) sequences (5).

**TABLE 1:** Results of the genomic sequencing of five RHDV2 strains detected in feral European rabbits found dead in Washington state in 2024.

WA/2024 RHDV2 strain	Sequencing instrument, kit	Read length (nt)	Library reads (M)	% of reads mapped	Consensus length (nt)	Consensus coverage (reads/nt)	GC%
SJ1	MiSeq, Reagent Kit v3 (600 cycles)	301	1.4	87.7	7,376	35,064	50.7
SJ2	NextSeq 1000, XLEAP-SBS P2 (600 cycles)	301	10.2	73.2	7,379	252,480	50.7
SJ3	NextSeq 1000, XLEAP-SBS P2 (600 cycles)	301	7.9	71.1	7,379	196,387	50.7
SJ4	NextSeq 1000, XLEAP-SBS P2 (600 cycles)	301	8.5	51.3	7,372	145,727	50.7
KC1	NextSeq 1000, XLEAP-SBS P2 (600 cycles)	301	4.2	59.2	7,375	87,208	50.7

**Fig 1:**
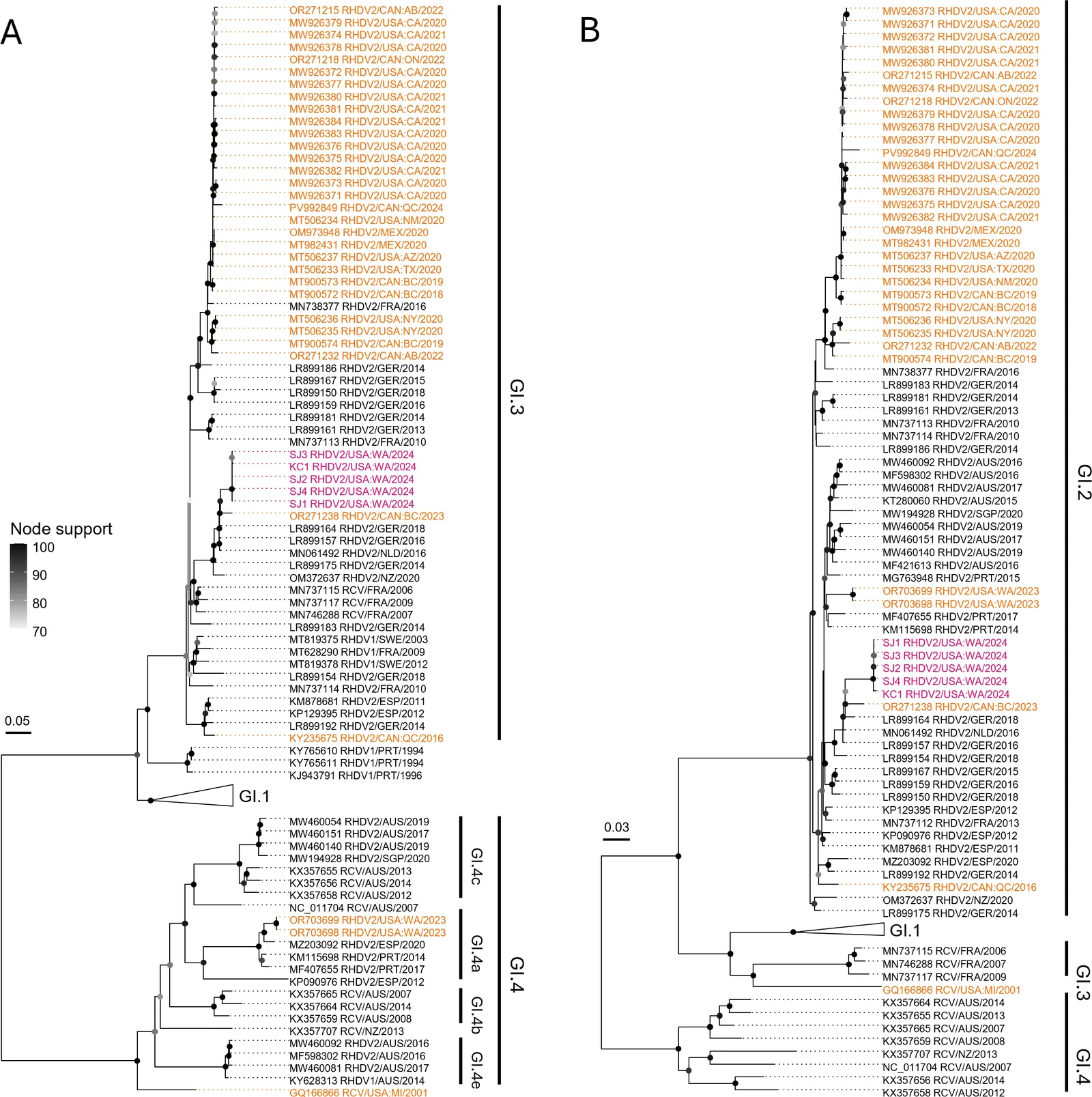
Maximum likelihood phylogenetic trees of (**A**) non-structural and (**B**) structural coding regions of GI lagoviruses generated using IQ-Tree2 2.3.5 with the best-fitted model determined by modeltest (SYM + R4 and SYM + I + G4, respectively) and 1,000 ultrafast bootstraps. North American sequences are highlighted in orange, with the five new WA strains in pink. Node points are colored based on node support. The GI.1 clade was collapsed for better visualization. Scale bars represent substitutions per site. Clades are labeled by vertical lines as previously described (5).

Maximum likelihood analyses supported the WA/2024 RHDV2 strains (SJ1-4, KC1) as each other’s closest relatives and a 2023 RHDV2 strain detected in Canada (RHDV2/CAN:BC/2023; OR271238.1) as the sister group (Fig. 1). These six NA RHDV2 strains were identified as genotype GI.3P-GI.2 with a close relationship to 2018 German strains (LR899164, LR899157) in both the S and NS gene trees. Interestingly, WA/2023 strains (OR703698, OR703699) were identified as GI.4aP-GI.2 with a close relationship to Portuguese (KM115698, MF407655) and Spanish (MZ203092) strains (6). Considering the absence of rabbit caliciviruses in NA that could serve as the donor for the NS genes in WA/2024 and given the <97.5% nucleotide identity of the S gene sequences, which exceeds the rate of evolution for the RHDV capsid gene (10), recombination of the WA/2023 strain resulting in WA/2024 is highly unlikely. Thus, our analysis supports independent incursions of the WA/2023 and WA/2024 strains into NA.

## Data Availability

Coding sequence complete genomes for each sample are available in GenBank under the following accession numbers: SJ1, PZ170256.1; SJ2, PZ170258.1; SJ3, PZ170257.1; SJ4, PZ170259.1; and KC1, PZ170260.1. The sequence reads for the project are available under BioProject accession number PRJNA1457561.
